# Patterns of AI Use in Clinical Work by Hospitalists: Survey Study

**DOI:** 10.2196/85973

**Published:** 2026-03-03

**Authors:** Prabhava Bagla, Jasmah Hanna, Bhargav Marthambadi, Stacey Watkins

**Affiliations:** 1 Department of Medicine, Division of Hospital Medicine School of Medicine Emory University Atlanta, GA United States; 2 Grady Memorial Hospital Atlanta, GA United States

**Keywords:** artificial intelligence, AI, generative artificial intelligence, generative AI, hospitalist, physician, patterns of use, clinical decision support systems, healthcare technology adoption

## Abstract

**Background:**

Artificial intelligence (AI) tools are widely and freely available for clinical use. Understanding hospitalists’ real-world adoption patterns in the absence of organizational endorsement is essential for health care institutions to develop governance frameworks and optimize AI integration.

**Objective:**

The objective of this study was to investigate hospitalists’ use of AI, examining the AI platforms being used, frequency of use, and clinical contexts of application. We hypothesized that AI use is more common among younger, less experienced hospitalists, albeit at an overall low frequency.

**Methods:**

An anonymous online survey was distributed via email to all 70 hospitalists (physicians, nurse practitioners, and physician assistants) providing direct patient care at a large urban academic tertiary care hospital. Demographic data, the AI platform used (if any), the purpose for AI use, and the frequency of use information were collected. The CHERRIES (Checklist for Reporting Results of Internet E-Surveys) checklist was used for creating, testing, administering, and reporting the results of the survey. Chi-square test was used where possible; when expected cell values were low, the Fisher's exact test was used instead. The Friedman test and the pairwise Wilcoxon signed-rank test were used for analyzing the differences in the frequency of AI use for various tasks. Likert-scale responses to frequency questions (never, rarely, sometimes, often, and always) were converted to ordinal values (1-5, respectively) to facilitate analysis.

**Results:**

Of the 70 providers, 54 (77.1%) responded to the survey. No significant differences in AI usage were observed across shift type, years of practice, time allocation to hospitalist duties, sex, age, or provider designation, contrary to our hypothesis. Overall, 36 of 54 respondents (66.7%; 95% CI 53.4%-77.8%) reported using AI in clinical practice. OpenEvidence was the most used platform (28/54, 51.9%), far exceeding general-purpose tools like OpenAI’s ChatGPT (4/54, 7.4%), suggesting a preference for medical-specific platforms. Among nonusers, primary concerns were AI accuracy and preference for established resources. The most common application was answering miscellaneous clinical questions (32/36, 88.9%), generating differential diagnoses (31/36, 86.1%), and determining management options (31/36, 86.1%), with much lower use for patient education materials (16/36, 44.4%). There was a statistically significant difference in the frequency of AI use across these clinical scenarios (Friedman test *χ*^2^_4_ 37.6; *P*<.001). Pairwise comparisons using the Wilcoxon signed-rank test revealed significant differences between use for answering miscellaneous questions and confirming suspected diagnosis (*P*=.003) and generating patient education materials (*P*=.004), respectively. Most respondents reported using AI for under 25% of clinical encounters across all use cases.

**Conclusions:**

Two-thirds of hospitalists organically adopted AI despite the absence of institutional oversight. AI is predominantly used as a supplementary decision support tool, with a preference for a medical-specific platform. Health care institutions must develop governance frameworks, validation protocols, and educational initiatives to ensure safe and effective AI deployment in clinical practice.

## Introduction

The development of artificial intelligence (AI) models has led to a significant interest in exploring their capabilities in many aspects of clinical medicine [1,2]. AI systems have been tested against physicians in assessing acuity [3], developing diagnostic formulation [4], and generating management options [5]. Particularly in image or pattern recognition-based diagnosis, AI demonstrates comparable or superior performance to clinicians [6,7].

While numerous studies have evaluated AI performance in carefully controlled research settings, large language model (LLM)–based AI tools are also widely and freely available without restriction to specific, well-defined tasks. An LLM is pretrained on a large amount of data, and can leverage that knowledge to answer user queries in natural language format. LLM capabilities range from presenting relevant information sourced from data it has access to, generating new content, such as computer code, or editing text [8]. ChatGPT is a general-purpose AI system offered by OpenAI that has a range of free and premium tier options, with progressively more advanced reasoning and content generation capabilities available [9]. A survey of US adults by the Pew Research Center indicated that between March 2023 and March 2025, awareness about ChatGPT has increased from 58% to 79%, and 28% of employed adults reported using it for work, compared with 8% in 2023. Usage rates are higher among younger adults and those with higher education levels, such as a postgraduate degree [10]. Some AI systems, such as Gemini by Google and Copilot by Microsoft, are presented to consumers by means of integration into their respective technical offerings, including prominently alongside the basic web search interface [11,12]; with free and paid user tiers and capabilities available similar to ChatGPT [13,14].

Some AI platforms use a retrieval-augmented generation (RAG) system for ease of updating and improved reliability of their LLM’s output. An RAG system provides a specific, reliable database for an LLM to search, in addition to what it has already been trained on. The LLM then combines its pre-existing knowledge and the information from the database to synthesize responses to user queries. The RAG strategy simplifies the process of keeping LLM answers updated and accurate in the context of evolving knowledge by simply expanding the authoritative database the LLM accesses, instead of having to retrain the LLM to search and appropriately parse all possible data sources on its own [15]. ClinicalKey AI is one such AI system developed by Elsevier requiring access using a paid subscription model that delivers evidence-based answers sourced from their library of books and journals [16].

OpenEvidence is a unique AI tool marketed specifically toward medical professionals only in the United States. It provides free unlimited use for verified users, while allowing some use with query limits to users without creating an account. It also uses the RAG system by means of data use agreements with several reputable sources, such as the *New England Journal of Medicine*, *Journal of the American Medical Association Network* journals, National Comprehensive Cancer Network, American Diabetes Association, and American College of Cardiology, and thus has the potential to offer evidence-based answers grounded in peer-reviewed medical literature. It also offers the ability to write patient handouts for education about management options for diseases, posthospitalization care, and insurance prior authorization letters [17].

AI tools have been implemented for use for a wide variety of purposes in health care, such as (1) clinical use by health care providers for supporting patient care decisions; (2) directly by laypersons for self-evaluation of health concerns; (3) facilitating business operations, such as bed management; and (4) as hybrid tools supporting both patient care and optimizing health care delivery, such as AI scribes for clinical documentation [18]. A study of 67 nonprofit health systems revealed adoption of AI for active or planned use mostly in imaging and radiology, followed by electronic health record integration for purposes like sepsis detection, and generating documentation via ambient listening [19].

An individual health care provider’s use of AI might be influenced by how others perceive them. A study on public perception of physicians who use AI, conducted by surveying US adults shown hypothetical static advertisements for family physicians differing in whether AI use for administrative, diagnostic, or therapeutic purposes was mentioned, showed that physicians using AI for any purpose were considered less competent, trustworthy, and empathic, as well as less sought after for care [20]. Similarly, practicing clinicians viewed hypothetical peers who used AI as a primary decision-making tool to be significantly less competent than those who did not use AI at all, an effect only partially mitigated if the use of AI was specified as being used for verification purposes [21].

Despite these perception issues, physicians’ sentiment toward AI use in clinical practice is overall positive, with greater familiarity, use in daily or professional life, and being involved in AI research as factors associated with greater enthusiasm and lower skepticism; the primary concerns being liability in the event of errors, and lack of transparency in AI decision-making [22]. The American Medical Association (AMA) conducted a survey of 1183 physicians, indicating that AI use increased from 38% in 2023 to 66% in 2024, mostly for assistance with documentation, ranging from discharge summaries to responses to patient portal messages. Respondents also indicated that measures to reduce administrative burden held the greatest promise for future AI implementation, and an increasing recognition of the potential of AI to benefit patient care (68% in 2024 compared with 63% in 2023) [23].

While adoption of AI at an organizational level leads to availability and use for preapproved tasks by health care providers working in the respective organizations, individual providers are unable to use AI for any tasks that require deep integration with their organization’s electronic health record system at a system level. Hospitalists represent a unique population for studying AI adoption given their role as frontline providers, managing high volumes of diverse, acutely ill patients across multiple clinical domains. Their practice patterns, workflow demands, and decision-making processes may influence how AI tools are integrated into daily clinical care. However, there is a dearth of literature about AI real-world usage patterns in daily clinical practice among hospitalists in the absence of any organizational endorsement.

The purpose of this study was to investigate hospitalists’ use of AI through a comprehensive survey examining the types of AI tools currently being used, frequency of use, and clinical contexts of application in real-world hospital practice settings without any system-wide AI integration. We hypothesized that AI use in clinical work is more common among younger, less experienced hospitalists, albeit at an overall low frequency.

## Methods

### Study Design

A comprehensive anonymous online survey (Multimedia Appendix 1) was distributed via email during August 2025 to all 70 physicians, nurse practitioners, and physician assistants (the latter 2 categories collectively referred to as advanced practice providers [APPs]) who provide direct patient care at our large, tertiary care academic center. The sample was not considered a convenience sample as all providers were given the opportunity to respond. The survey asked demographic questions and questions about which AI systems, if any, the respondents used and for what purpose. For analytic purposes, AI platforms were categorized as either general-purpose or medical-specific based on their primary market positioning and intended use at the time of the survey. Medical-specific platforms were defined as tools marketed explicitly for clinical decision support and designed primarily for use by health care professionals, with content grounded in medical literature or curated clinical sources (OpenEvidence and ClinicalKey AI). General-purpose platforms were defined as broadly available AI tools intended for use across multiple domains and audiences, not marketed primarily for clinical decision-making (ChatGPT, Microsoft Copilot, and Google Gemini), even if health care–specific enterprise or specialized versions may exist.

If respondents indicated that they did not use any AI systems, they were also asked what concerns they had. CHERRIES (Checklist for Reporting Results of Internet E-Surveys) guidelines (Multimedia Appendix 2) on survey studies were followed [24].

### Ethical Considerations

This study was considered not to comprise human participant research by Emory University Institutional Review Board in accordance with the Declaration of Helsinki, and so was exempt from full committee review. The first page of the survey contained information regarding the purpose of the study, potential risks, and benefits to respondents, and contact information regarding the investigators. If respondents did not wish to answer the survey, they could either decline using the option provided or simply close the survey. The page also disclosed that response data would be kept confidential, only analyzed as an aggregate, and the information stored securely without any specific time limits using an encrypted institutional subscription to Microsoft OneDrive and a related set of tools. No personally identifiable information was collected as part of the survey. No compensation or incentive of any kind, monetary or otherwise, was offered to participants.

### Survey Development

Survey questions were developed by discussion and consensus among study authors. For AI users, the survey asked, “How often, on average, do you use AI per day for [specific clinical task]?” for each of five tasks, (1) generating differential diagnosis lists, (2) confirming suspected diagnosis, (3) answering miscellaneous questions (with examples provided, such as rare drug side effects), (4) determining management or testing options, and (5) generating patient education materials. Response options were never, rarely, sometimes, often, and always; defined as (1) rarely (0%-25% of the time), (2) sometimes (26%-50% of the time), (3) often (51%-75% of the time), and (4) always (76% or more of the time). The survey did not explicitly specify whether respondents should consider all patient encounters or only those encounters where the specific task would be applicable, leaving interpretation to individual respondents. Functionality was tested before dissemination to potential respondents.

### Recruitment Process

The survey was distributed via email during August 2025 to all 70 potential respondents. They were subsequently reminded over a period of 3 weeks via group emails and in-person interactions.

### Survey Administration

The email sent to potential respondents contained a brief description of the study and a link to the survey itself. The survey was created in Microsoft Forms, which allowed responses to be automatically saved as a Microsoft Excel spreadsheet. Adaptive questioning was used to streamline survey responses and navigation; for instance, if respondents indicated they did not use AI for clinical work at all, they were not shown questions about possible use scenarios. The maximum number of question items (including initial informed consent) was 17, with an average of 2 questions per page for readability. All questions were marked mandatory, so incomplete responses were not allowed. All questions, except for reasons for not using AI at all, were single-response only, and nonresponse options were provided for some of the demographic questions. Respondents could adjust their responses before submitting, at which time further editing of responses was not allowed. No incentives were offered in exchange for responses. Overall, responses were collected during August-September 2025.

### Response Rates

Unique responses were ensured by requiring respondents to log in (automatically fulfilled when accessing the survey by clicking the link in their email) and only allowing 1 response per person. We are unable to determine if there were potential respondents who viewed the survey but did not answer any questions or stopped partway, as only completed surveys were accepted.

### Statistical Analysis

Statistical analysis was conducted using SAS (version 9.4, SAS Institute Inc) for analysis of demographic variables. Chi-square test was used where possible; when more than 20% of expected cell values were under 5, the Fisher's exact test was used instead. R software (version 4.5.1; R Foundation) was used for the Friedman test and the pairwise Wilcoxon signed-rank test for analyzing the differences between the frequency of AI use for various tasks. These analyses were restricted to responses to survey questions asking about the 5 standardized clinical tasks (generating differential diagnosis lists, confirming suspected diagnosis, answering miscellaneous questions, determining management or testing options, and generating patient education materials; Multimedia Appendix 1). Likert-scale responses to survey questions (never, rarely, sometimes, often, and always) were converted to ordinal values (1-5, respectively) to facilitate analysis. Only completed surveys were analyzed. No specific analysis regarding the time taken to complete the survey was conducted, and no weighting or propensity scores were used.

## Results

Out of 70 providers, 54 responded to the survey (response rate of 77.1%). Table 1 presents survey respondent characteristics and AI usage patterns. The majority of respondents were dayshift team members (48/54, 88.9%), and two-thirds (36/54, 66.7%) were within 6 years of completing training. Most respondents devoted all their time to clinical work (31/54, 57.4%). Physicians comprised 83.3% (45/54) of respondents, with APPs accounting for the remaining 16.7% (9/54). No statistically significant differences in AI usage were observed across shift type, years of practice, time allocation to hospitalist duties, sex, age, or provider designation, contrary to our hypothesis that younger, less experienced hospitalists would use AI more frequently. Ethnicity was not analyzed for statistical significance due to insufficient sample sizes across groups. Overall, 36 of 54 (66.7%; 95% CI 53.4%-77.8%) respondents reported using AI tools in clinical practice.

**Table 1:** Characteristics of survey respondents and association with artificial intelligence use in clinical work (n=54). Survey respondents from a large academic medical center, August-September 2025.

Variable		Number (n=54), n (%)	Use of AI^a^, n (%)	*P* value^b^	
**Role**					>.99^b^
	Dayshift rounding and admissions	48 (88.9)	32 (66.6)		
	Night shift admissions and cross-cover	6 (11.1)	4 (66.6)		
**Years of practice (y)**					.54
	0-6	36 (66.7)	25 (69.4)		
	7 or more	18 (33.3)	11 (61.1)		
**Amount of time spent on clinical hospitalist duties**					.12
	100%	31 (57.4)	18 (50.6)		
	99% or less	23 (42.6)	18 (78.3)		
**Sex**					.44
	Female	26 (48.1)	16 (61.5)		
	Male	28 (51.9)	20 (71.4)		
**Age (y)**					.11
	35 or less	20 (37)	16 (80)		
	36 or more	34 (63)	20 (58.8)		
**Ethnicity^c^**					—^d^
	Asian	15 (27.8)	10 (66.6)		
	Black or African American	12 (22.2)	8 (66.6)		
	Hispanic or Latino	3 (5.6)	3 (100)		
	White	20 (37)	12 (60)		
	Prefer not to say	4 (7.4)	3 (75)		
**Designation**					>.99^b^
	Physician	45 (83.3)	30 (66.6)		
	APP^e^	9 (16.7)	6 (66.6)		
**Use of AI in clinical work**					—
	Yes	36 (66.7)	—		
	No	18 (33.3)	—		

^a^AI: artificial intelligence.

^b^Fisher's exact test was used when specified, otherwise chi-square test was used.

^c^Statistical analysis not performed due to low sample sizes.

^d^Not applicable.

^e^APP: advanced practice provider.

OpenEvidence was the most commonly used platform (28/54, 51.9%), followed by ChatGPT (4/54, 7.4%; Table 2; Multimedia Appendix 3). Among the 18 respondents (33.3%) who did not use AI, the barriers to adoption were (1) preference for alternative resources such as PubMed (14/18, 77.8%); (2) concerns regarding accuracy (7/18, 38.9%); (3) not available system-wide, or endorsed by hospital, or via the electronic medical record (7/18, 38.9%; respondents who answered not available system-wide or endorsed by hospital also selected not available via electronic medical record); (4) time constraints (4/18, 22.2%); (5) privacy concerns (4/18, 22.2%); (6) prefer to consult another provider (2/18, 11.1%); and (7) lack of continuing medical education credits (1/18, 5.6%); no respondents selected cost or weekly query limits as reasons (percentages do not add up to 100% as multiple responses were allowed for this question). The question also allowed for free-text input of other reasons that respondents did not use AI, but no additional responses were received.

**Table 2:** Artificial intelligence platforms used by hospitalists in clinical work (n=54). Survey respondents from a large academic medical center, August-September 2025.

Use of AI^a^ in clinical work	Number (n=54), n (%)
Microsoft Copilot	1 (1.9)
Google Gemini	1 (1.9)
ClinicalKey AI	2 (3.7)
ChatGPT	4 (7.4)
OpenEvidence	28 (51.9)
No use of any AI	18 (33.3)

^a^AI: artificial intelligence.

Table 3 and Multimedia Appendix 4 summarize the clinical tasks for which AI was used by users (n=36). The most common application was answering miscellaneous clinical questions (32/36, 88.9%; 95% CI 74.7%-95.6%), with respondents providing examples, including “rare drug side effects” and “causes of false-positive urine drug screen.” Generating differential diagnoses and determining management or treatment options were equally prevalent (31/36, 86.1%; 95% CI 71.3%-93.9%). Approximately 72.2% (26/36; 95% CI 56%-84.2%) of responders used AI to support confirmation of suspected diagnoses, while few (16/36, 44.4%; 95% CI 29.5%-60.4%) used AI for generating patient education or counseling materials. Using the Friedman test revealed there was a statistically significant difference in the frequency of AI use across these clinical scenarios (*χ*^2^_4_=37.6; *P*<.001).

**Table 3:** Clinical tasks for which artificial intelligence is used among hospitalist AI users (n=36). Survey respondents from a large academic medical center, August-September 2025.

Tasks AI^a^ is used for	Number (n=36), n (%)	95% CI
Generating differential diagnosis list	31 (86.1)	71.3%-93.9%
Confirming suspected diagnosis	26 (72.2)	56%-84.2%
Answering miscellaneous questions	32 (88.9)	74.7%-95.6%
Determining testing or treatment options	31 (86.1)	71.3%-93.9%
Generating patient education or counseling materials	16 (44.4)	29.5%-60.4%

^a^AI: artificial intelligence.

Pairwise Wilcoxon signed-rank test with continuity and Bonferroni corrections demonstrated that AI use for answering miscellaneous questions was significantly different from use for confirming suspected diagnosis (corrected *P*=.003) and generating patient education or counseling materials (corrected *P*=.004).

Table 4 details the frequency of AI usage for each clinical application. Respondents were asked how often, on average, they used AI per day for each specific task. Most respondents reported using AI rarely (0%-25% of the time) across all use cases, in line with our hypothesis. Additionally, 3 respondents indicated other AI applications—assistance with clinical documentation, comprehensive literature searches, and creation of educational cases for medical students (1 respondent for each).

**Table 4:** Frequency of artificial intelligence use for specific clinical tasks among hospitalist artificial intelligence users (n=36). Survey respondents from a large academic medical center, August-September 2025.

Tasks^a^ AI^b^ is used for		Number (n=36), n (%)
**Generating differential diagnosis list**		
	Never	5 (13.9)
	Rarely	20 (55.6)
	Sometimes	8 (22.2)
	Often	2 (5.6)
	Always	1 (2.8)
**Confirming suspected diagnosis**		
	Never	10 (27.8)
	Rarely	17 (47.2)
	Sometimes	7 (19.4)
	Often	1 (2.8)
	Always	1 (2.8)
**Answering miscellaneous questions**		
	Never	4 (11.1)
	Rarely	12 (33.3)
	Sometimes	12 (33.3)
	Often	6 (16.7)
	Always	2 (5.6)
**Determining testing or treatment options**		
	Never	5 (13.9)
	Rarely	20 (55.6)
	Sometimes	4 (11.1)
	Often	5 (13.9)
	Always	2 (5.6)
**Generating patient education or counseling materials**		
	Never	20 (55.6)
	Rarely	9 (25)
	Sometimes	3 (8.3)
	Often	3 (8.3)
	Always	1 (2.8)
**Other purpose, not specified above**		
	No	33 (91.7)
	Yes	3 (8.3)

^a^Respondents were asked, “How often, on average, do you use AI per day for [specific task]?” Rarely: 0%-25% of the time; Sometimes: 26%-50% of the time; Often: 51%-75% of the time; Always: 76% or more of the time.

^b^AI: artificial intelligence.

## Discussion

### Principal Findings

Our findings reveal that two-thirds of hospitalists at our institution use AI tools during clinical work, predominantly for answering miscellaneous clinical questions (32/36, 88.9%), generating differential diagnoses (31/36, 86.1%), and determining management options (31/36, 86.1%). Notably, this usage occurs despite the absence of system-level integration, institutional endorsement, or selective blocking of any AI platform interface, suggesting organic adoption driven by perceived clinical utility. The predominance of OpenEvidence among AI users (28/36, 77.8% users; 28/54, 51.9% of all respondents) is particularly noteworthy. The high degree of convergence on this single niche platform, rather than more widely known general-purpose tools, raises questions about adoption pathways that warrant further investigation. The stark preference for specialized, medical-specific platforms (as defined by primary market positioning and intended clinical use) over general-purpose AI represents one of our most significant findings. Among AI users, OpenEvidence was chosen 7 times more frequently than ChatGPT (28 vs 4 respondents), despite ChatGPT’s substantially greater name recognition and broader public adoption [10]. This pattern suggests that hospitalists are not simply adopting whatever AI tools are most readily available or widely marketed, but are instead making deliberate choices based on tool-specific characteristics relevant to medical practice.

Differential availability of various AI platforms could have been a confounding variable affecting observed preference for one platform over others. For instance, if hospital firewall systems blocked access to general-purpose AI while allowing medical use–focused platforms, that could affect adoption rates. However, this was not a factor in our study as our institution’s computer systems do not block any particular AI platform.

Several factors may explain this preference for medical-specific platforms. First, hospitalists may perceive that specialized tools trained on or linked to medical literature will provide more accurate and relevant clinical information. Second, the use of general-purpose AI for patient care may raise concerns about data privacy, lack of possibility of HIPAA (Health Insurance Portability and Accountability Act) compliance, and liability that are less salient with tools explicitly designed for health care applications. Third, medical-specific platforms may provide source citations or references to primary literature, offering a verification pathway that general-purpose tools typically lack. Finally, hospitalists may view general-purpose AI as insufficiently tailored to the complexity and nuance of clinical decision-making, preferring tools that understand medical context and terminology.

This finding has important implications for AI development and deployment in health care. It suggests that clinicians value specialized tools over general-purpose solutions. Published literature validating OpenEvidence’s capabilities as a clinical decision support tool for use by health care providers remains limited to generating management options for hypothetical primary care scenarios [25]; yet, hospitalists have adopted it widely based on its medical focus alone. This creates both an opportunity and a responsibility for developers of medical-specific AI tools to ensure their platforms meet the quality and safety standards that clinicians appear to assume they possess. The preference for medical-specific platforms also highlights a potential vulnerability that clinicians may place unwarranted trust in tools marketed as “medical AI” without rigorous independent validation of their accuracy and reliability compared with general-purpose alternatives.

The relatively low frequency of AI use, with most respondents reporting use in 0%-25% of their daily work, suggests that AI currently serves as a supplementary rather than primary clinical resource. Importantly, the survey did not specify whether frequency estimates should be calculated using all daily patient encounters or only those encounters in which the task was relevant. As a result, responses categorized as “rarely” (0%-25%) could reflect near-universal AI use for a small subset of high-acuity or diagnostically complex cases. This ambiguity limits the precision of our frequency estimates and necessitates caution when interpreting AI use as uniformly “low” or purely supplementary. We hypothesize that hospitalists are selectively deploying AI for cases where traditional resources such as UpToDate or PubMed searches are perceived as insufficient or time-intensive. The statistically significant differences in AI usage across clinical applications provide insight into how hospitalists perceive AI’s strengths and limitations. The high usage for answering specific clinical questions, generating differential diagnoses, contrasted with lower usage for patient education materials (16/36, 44.4%), may reflect concerns about accuracy and liability when AI-generated content interfaces directly with patients, demonstrating appropriate professional judgment in the face of evolving technology, although it may also reflect a perception of low workflow efficiency in the setting of lack of institutional integration of AI.

Among nonusers (18/54, 33.3%), the primary concerns centered on preference for established resources, AI accuracy, and system-wide endorsement, followed by time constraints and questions about privacy. These findings highlight important barriers that must be addressed for broader adoption, such as validation of AI outputs, integration with existing workflows, and clear guidance on appropriate use cases. The absence of statistically significant demographic predictors of AI adoption suggests that usage patterns may be driven more by individual preferences and comfort with technology than by systematic factors, such as years in practice or clinical role.

### Comparison With Previous Work

Purpose-built AI systems have been integrated into clinical workflows for narrowly defined tasks, such as radiologic interpretation [1,2], and experimental studies have compared AI performance against physicians in diagnostic and management scenarios [4-7]. However, adoption patterns of freely available AI tools by health care providers in the absence of institutional integration have remained largely uncharacterized. Our study addresses this knowledge gap by documenting how, and for what purposes hospitalists are independently incorporating AI into their daily practice and for what purposes.

Our observed adoption rate of 66.7% (95% CI 53.4%-77.8%) is comparable to the 66% reported in the 2024 AMA physician survey [23], suggesting that individual adoption patterns may mirror broader trends even without institutional mandates. However, the contexts and applications differ substantially. The AMA survey found that AI was used predominantly for administrative tasks, particularly clinical documentation ranging from discharge summaries to patient portal message responses [23]. Poon et al [19] found health systems primarily implementing AI for imaging or radiology, sepsis detection, and ambient listening clinical documentation. In contrast, our respondents used AI primarily for clinical decision support—answering medical questions, generating differential diagnoses, and determining management options, although OpenEvidence, the platform chosen by a majority of our respondents, offers the capability to generate documentation meant for patient and health insurance use. This difference likely reflects the nature of institutional versus individual adoption—health systems implementing AI at an organizational level appear to prioritize electronic health record integration and workflow efficiency, whereas individual clinicians selecting their own tools focus on addressing clinical uncertainties and supplementing their decision-making process. Additionally, attempts by health care providers to use AI for documentation without pre-existing systematic integration involve extra steps (such as copy-pasting to and from AI platforms), which may also hinder adoption for such purposes. This distinction has important implications for how we conceptualize AI adoption; organizational implementation focuses on efficiency and administrative burden reduction, while individual adoption focuses on enhancing clinical reasoning and knowledge access.

Among nonusers (18/54, 33.3%) in our study, the primary concerns centered on AI accuracy and preference for established resources, consistent with the findings of Heinrichs et al [22] regarding physician skepticism about AI reliability. Interestingly, none of our respondents cited public or peer perception as factors influencing their decision not to use AI, despite recent studies demonstrating significant perception-based concerns. Reis et al [20] found that US adults rated physicians who used AI as less competent, trustworthy, and empathic, while Yang et al [21] showed that clinicians viewed peers using AI for primary decision-making as significantly less competent. The absence of perception concerns in our sample may reflect several possibilities, such as (1) hospitalists may be unaware of these negative perceptions, (2) they may not consider patient or peer opinions as relevant to their personal tool selection, (3) the anonymous nature of AI use—when used as a supplementary resource consulted privately rather than a visible component of patient-facing care—may shield providers from perception-based concerns, or (4) the lack of perception concerns as a selectable checkbox answer in the question led to the survey not capturing this as a reason, although there was a free-text option for those who wished to provide additional reasons. The discrepancy warrants further investigation, as understanding barriers to AI adoption—whether accuracy-based or perception-based—is essential for developing appropriate governance frameworks and educational initiatives.

The preference for medical-specific platforms observed in our study represents a novel finding not previously documented in the literature. While general-purpose AI tools, like ChatGPT, have achieved widespread adoption among employed adults [10], our hospitalists demonstrated a marked preference for domain-specific tools, with OpenEvidence accounting for the majority of AI users. This suggests that when clinicians independently select AI tools for clinical applications, they prioritize a medical focus and source transparency over general capability or familiarity. Whether this preference is justified by the superior performance of medical-specific platforms remains a question requiring comparative validation studies.

### Implications

Understanding current AI usage patterns has significant implications for health care institutions, educators, and policymakers. The widespread organic adoption of AI tools in the absence of institutional oversight underscores an urgent need for governance frameworks that balance innovation with patient safety. Health care systems should consider developing policies that guide appropriate AI use, establish standards for tool selection, and provide training on recognizing AI limitations. As health care systems increasingly invest in AI technologies, empirical data on actual usage patterns become essential for resource allocation and strategic planning. Rather than implementing AI systems that remain unused, institutions can leverage usage data to identify high-value applications that align with clinician workflows and needs. Furthermore, medical education must evolve to prepare trainees for AI-augmented clinical practice, including teaching critical appraisal of AI outputs, understanding the underlying technology and its limitations, and developing frameworks for integrating AI responsibly into clinical decision-making. The convergence on a single platform among users in our study highlights the importance of understanding AI adoption pathways. Whether through peer-to-peer sharing, targeted marketing, or other mechanisms, clinicians are selecting specific tools in ways that may not be captured by traditional technology adoption models. Health care institutions should recognize that even in the absence of formal mandates, departmental culture and informal peer networks likely shape which AI tools gain traction, underscoring the need for governance frameworks that can adapt to grassroots adoption patterns.

The strong preference for a medical-specific AI platform over general-purpose tools observed in our study suggests that clinicians are not passive recipients of whatever AI technology becomes available but are actively selecting tools based on perceived fit with medical practice. This preference may reflect appropriate professional judgment about the importance of medical domain knowledge, source transparency, and health care–specific design. Health care institutions and regulatory bodies should recognize that clinicians’ preference for specialized tools creates a need for rigorous, independent validation of these platforms’ accuracy, reliability, and safety.

A critical consideration in AI adoption is the dynamic nature of these systems. AI platforms undergo frequent updates by their manufacturers; yet, each iteration does not necessarily represent an improvement over its predecessor [26]. Unlike static clinical resources, such as textbooks or established guidelines, AI systems may change their outputs without notification to users, in the form of variation in accuracy of answers, and ability to perform tasks, such as following multistep questioning between models and even over time within the same model [27], introducing uncertainty into clinical application. As AI use in health care increases, governance frameworks for maintaining patient safety need to be put in place. Zhang and Zhang [28] identified challenges that impact individual and organization trust in AI, including concerns about the quality of data used to train AI systems, accuracy of the underlying algorithms, robustness of health care data protection, responsibility for actions based on AI results, and lack of full transparency and understanding about the inner workings of how AI produces results from data input [28]. There is no broad regulatory oversight of AI use in health care at present. In the United States, the Food and Drug Administration regulates only some AI systems, such as purpose-built AI for use on medical devices like defibrillators [29].

Given these challenges, primary source verification remains essential when using AI in clinical practice. We recommend that clinicians treat AI-generated information as a starting point requiring confirmation rather than a definitive answer. This principle is particularly important for hospitalists managing acutely ill patients, where diagnostic or therapeutic errors can have immediate consequences.

### Limitations

This study has several limitations. As a single-center investigation conducted at a large academic tertiary care hospital, our findings may not be generalizable to community hospitals, rural settings, or outpatient practice environments, where clinical workflows and case complexity differ substantially. The cross-sectional design provides a snapshot of current practice but cannot capture temporal trends or the trajectory of AI adoption. Self-reported usage data may be subject to recall bias, social desirability bias, or selection bias, with respondents possibly more technologically inclined than nonrespondents. Notably, our calculated adoption rate of 66.7% is likely an overestimate of the true institutional adoption rate, as nonusers of AI may have been less motivated to complete a voluntary survey about AI usage patterns compared with enthusiastic users. Additionally, this rate includes respondents across the spectrum of frequency of use, ranging from those who only rarely use AI to power users who use AI much more often. We were unable to directly compare every aspect of respondent demographics with those of the full hospitalist provider group, as detailed information regarding some categories, such as age and experience levels, is not available for nonrespondents. Although the overall response rate was high, residual nonresponse bias cannot be fully excluded. Although no statistically significant demographic predictors of AI use were identified, the relatively small number of AI users (n=36) and limited subgroup sizes (for instance, physicians vs APP) reduce statistical power. As such, the absence of detected associations should be interpreted as the absence of evidence rather than evidence of absence. Another minor limitation is the possible selection bias introduced by our survey design, which required responses (even if selecting a nonresponse option where available) to all questions. Our survey did not undergo formal pilot testing before wide dissemination, possibly having led to some ambiguities. Our frequency estimates asked respondents how often they used AI “per day” for each task without specifying whether to consider all patient encounters or only those where the task would be applicable. For instance, when rating frequency of AI use for generating differential diagnoses, some respondents may have considered all daily patient encounters as the denominator, while others may have mentally restricted it to encounters with diagnostic uncertainty. This ambiguity limits the precision of our frequency estimates, although the data clearly indicate that AI is used infrequently across all measured applications. There was some conceptual overlap between the provided choices of “never” and “rarely (0%-25%)” frequency categories, which could also affect our frequency estimates, although we expect that respondents who never use AI for a specific task, such as for generating a differential diagnosis list, would select “never” over “rarely” as an option. Also, our survey asked respondents for years of practice, which some may have interpreted to include training years, possibly confounding the analysis of experience levels. The survey instruction for years of practice asked respondents to round up to the nearest whole year, which may have resulted in potentially shifting some respondents into higher experience brackets. Although respondents were provided with a free-text option to report additional AI use cases, clinical documentation (separate from generating patient education materials) was not included as one of the predefined questions, which could have led to underreporting compared with tasks explicitly prompted in the survey. This could have contributed to the lower observed prevalence of documentation-related AI use relative to the AMA physician survey, in addition to workflow friction and lack of electronic health record integration. We did not collect data on the accuracy of AI outputs in actual clinical use or on whether AI recommendations were followed, limiting our ability to assess the impact of AI on clinical decision-making. Additionally, we only asked respondents which AI platform (if any) they used most often, and did not collect data on whether they were using more than 1 system. The preference for OpenEvidence may reflect a pattern of primary use (with additional tools used for other scenarios) rather than confirmation of exclusive use.

An important limitation is our lack of data regarding how respondents discovered and selected their AI tools. The striking convergence on OpenEvidence is noteworthy given its status as a niche platform compared with widely known tools, like ChatGPT. Also, although ClinicalKey AI is also a medical-specific platform, its adoption remained low. To our knowledge, there was no formal departmental introduction or recommendation of any platform, although informal peer-to-peer recommendations may have occurred through channels we did not measure. Without systematic data on discovery pathways, we cannot determine whether this convergence reflects informal sharing within our department, the platform’s targeted marketing toward medical professionals, lack of cost barriers, or other factors. While our institution does not block online access to any particular AI platform, other organizations may allow differential access to some systems versus others, which could affect local adoption patterns. Future studies should systematically assess how clinicians learn about and select AI tools, as understanding these adoption pathways has important implications for dissemination strategies and governance frameworks.

Furthermore, our survey did not capture the specific clinical scenarios or patient characteristics that prompted AI use. Despite these limitations, our study has notable strengths. The high response rate (77.1%) enhances the reliability of our findings and minimizes nonresponse bias. The comprehensive assessment of multiple AI platforms and diverse clinical applications they are being used for provides a nuanced picture of real-world usage. Our study to systematically document hospitalist AI usage patterns independent of organizational endorsement establishes baseline data for future longitudinal investigations and comparative studies across institutions and specialties.

### Future Directions

These findings point to several important directions for future research. Longitudinal studies are needed to track how AI usage patterns evolve as these technologies mature and as clinicians gain more experience with them. Comparative effectiveness research should evaluate clinical outcomes associated with AI-augmented versus traditional clinical decision-making. Additionally, research should examine whether AI usage varies across different clinical contexts, patient populations, and decision types, as understanding these nuances could inform the development of more targeted AI applications that address specific clinical needs. This includes future multicenter studies, which should explore interinstitutional variation and correlate AI adoption pathways and usage with clinical outcomes. Ongoing validation studies must continually assess AI system performance across the tasks they are commonly used for, with particular attention to detecting degradation or drift in performance over time. Public and peer-perception–based barriers to AI adoption also warrant further exploration. Finally, comparative studies examining the accuracy, reliability, and clinical utility of medical-specific AI platforms versus general-purpose LLMs for clinical applications would help determine whether the observed preference for specialized tools is justified by superior performance.

### Conclusions

This study documents substantial organic adoption of AI tools among hospitalists, with two-thirds reporting clinical use despite the absence of institutional integration or endorsement. The predominant applications—answering clinical questions, generating differential diagnoses, and determining management options—suggest that AI is serving as a supplementary decision support tool for complex cases. The marked preference for a medical-specific platform over a general-purpose AI indicates that hospitalists are exercising discretion in tool selection. This finding suggests both appropriate caution and a need for rigorous validation of medical-specific platforms. However, concerns about accuracy and the low frequency of use highlight the nascent state of AI integration in clinical practice. As AI technologies continue to evolve, health care institutions must develop governance frameworks, validation protocols, and educational initiatives to ensure these tools are deployed safely and effectively in patient care.

## Appendix

Multimedia Appendix 1 Copy of the survey questionnaire.

Multimedia Appendix 2 CHERRIES checklist.

Multimedia Appendix 3 Distribution of artificial intelligence platforms used by hospitalists in clinical work (n=54). Survey respondents from a large academic medical center, August-September 2025.

Multimedia Appendix 4 Clinical tasks for which artificial intelligence is used among hospitalist artificial intelligence users (n=36). Survey respondents from a large academic medical center, August-September 2025.

## Abbreviations

AI: artificial intelligence
AMA: American Medical Association
APP: advanced practice provider
CHERRIES: Checklist for Reporting Results of Internet E-Surveys
HIPAA: Health Insurance Portability and Accountability Act
LLM: large language model
RAG: retrieval-augmented generation

## Data Availability

The datasets generated or analyzed during this study are available from the corresponding author on reasonable request.
