# Supplementary material for: Patterns of AI Use in Clinical Work by Hospitalists: Survey Study
Source: J Med Internet Res. 2026 Mar 3;28:e85973. doi: 10.2196/85973 (PMC12996894; doi:10.2196/85973)
Supplement: Multimedia Appendix 1 [file jmir_v28i1e85973_app1.docx]

* Required

* 1. You Are Being Asked to Be in a Survey Study

We request that you answer a short online survey regarding use of Artificial Intelligence (AI) in clinical work.

- Why is this study being done?
- This study is being done to answer the question: what is the current pattern of use of AI by hospitalists in clinical work. You are being asked to be in this study because you are one of the hospitalists in the institution in which this pilot study is being conducted.
- Do you have to be in the study?
- It is your choice to join this study. You do not have to be in it. The survey is completely anonymous, and none of your personal details will be collected.
- What do you have to do if you choose to join this study?
- If you choose to participate in the study, you will fill out a short online anonymous survey that follows this form. By proceeding to the survey, you give your consent to participate.
- How is this study going to help you?
- If you are in the study, you will be helping the researchers answer the study question. This study is not intended to benefit you directly.
- What are the risks or discomforts you should know about before deciding?
- Your responses to the survey questions will be kept completely confidential, and will only be analyzed as an aggregate. No identifying information directly linking you to your responses is being asked for by the study team. We ask that you refrain from including any personally identifying information in your responses.
- Alternatives to Joining This Study
- Should you choose not to participate in this study, simply click “decline” at the bottom of this form.
- Costs
- There will be no costs to you for participating in this study
- What Should You Do Next?
- If you choose to participate in this study, please click “accept” at the bottom of this form to start the survey. If you have any further questions, please do not hesitate to contact us:

(1) Prabhava Bagla, MD

prabhava.bagla@emory.edu

(2) Bobby Marthambadi, MD

bhargav.s.marthambadi@emory.edu

(3) Jasmah Hanna, MS

jasmah.hanna@emory.edu

(4) Stacey Watkins, MD, PhD

stacey.michelle.watkins@emory.edu

* 2. Which of these best describes your primary clinical responsibility?

- Inpatient rounding +/- admissions (regular team)
- Day admitting and cross-covering (admitter/swing)
- Nocturnist admitting and cross-covering

* 3. How many years have you been practicing? (Please round up to nearest whole year)

- 0 - 3
- 4 - 6
- 7 - 10
- 11 years or more

* 4. What is your current hospitalist clinical FTE?

- 1.0
- 0.75 - 0.99
- 0.5 - 0.74
- 0.25 - 0.49
- Less than 0.24

* 5. If you use any AI system as part of clinical work, which do you use most frequently?

- ChatGPT
- Microsoft Copilot
- Google Gemini
- OpenEvidence
- ClinicalKey AI
- Other
- I do not use AI at all

**if survey responder selects “ChatGPT”, “Microsoft Copilot”, Google Gemini”, “OpenEvidence” or “ClinicalKey AI” for Question 5, survey responder will immediately proceed to Question 6.

* 6. How often, on an average, do you use AI per day for: generating differential diagnosis list?

- Never
- Rarely (0 - 25% encounters)
- Sometimes (26 - 50% encounters)
- Often (51 - 75% encounters)
- Always (76% or more encounters)

* 7. How often, on an average, do you use AI per day as part of clinical work for: confirming your suspected diagnosis?

- Never
- Rarely (0 - 25% encounters)
- Sometimes (26 - 50% encounters)
- Often (51 - 75% encounters)
- Always (76% or more encounters)

* 8. How often, on an average, do you use AI per day as part of clinical work for: answering miscellaneous questions? (eg rare drug side effects, or causes of false-positive urine drug screen results)

- Never
- Rarely (0 - 25% encounters)
- Sometimes (26 - 50% encounters)
- Often (51 - 75% encounters)
- Always (76% or more encounters)

* 9. How often, on an average, do you use per day AI as part of clinical work for: determining management (testing/treatment) options?

- Never
- Rarely (0 - 25% encounters)
- Sometimes (26 - 50% encounters)
- Often (51 - 75% encounters)
- Always (76% or more encounters)

* 10. How often, on an average, do you use AI per day as part of clinical work for: generating patient education/counseling materials?

- Never
- Rarely (0 - 25% encounters)
- Sometimes (26 - 50% encounters)
- Often (51 - 75% encounters)
- Always (76% or more encounters)

* 11. Do you use AI as part of clinical work for another purpose not specified above?

- Yes
- No

*12 What is your gender?

- Male
- Female
- Non-binary
- Prefer not to say

* 13. What is your age in years? (Please round up to the nearest whole year)

- 25 or younger
- 26 - 30
- 31 - 35
- 36 - 40
- 41 - 45
- 46 or older

* 14. What is your ethnicity?

- American Indian/Alaskan Native
- Asian
- Black or African-American
- Hispanic or Latino
- Native Hawaiian or other Pacific Islander
- White (European, Middle Eastern, other)
- Other
- Prefer not to say

* 15. Please specify your designation

- Physician
- APP

If survey responder selected “Other” for Question 5, survey responder proceeded to this additional question”

* Please specify

Enter your answer (free text answer box available)

From this question, survey responder continued with questions 6-15

If survey responder selected “I do not use AI at all” for Question 5, survey responder proceeded to this additional question:

*If you do not use any AI system in clinical work at all, why not? (please select all that apply)

- Concerned about accuracy
- Not available system-wide/endorsed by hospital
- Not available via Epic
- Time consuming
- Use of other resources (such as UpToDate, PubMed)
- Lack of CME credit from use
- Prefer to consult another provider
- Privacy concerns (eg. HIPAA)
- Cost of creating a personal account
- Weekly query limits without creating an account
- Other

If survey responder selected “Other” for the question above, survey responder proceeded to this additional question:

* Please specify

Enter your answer (free text answer box available)

Following this, survey responder continued with questions 12-15

If survey responder answered “Yes” to question 11., survey responder proceeded to this additional question:

*Please specify

Enter your answer (free text answer box available)

Following this, proceeded to questions 12 - 15
