# Supplementary material for: Patterns of AI Use in Clinical Work by Hospitalists: Survey Study
Source: J Med Internet Res. 2026 Mar 3;28:e85973. doi: 10.2196/85973 (PMC12996894; doi:10.2196/85973)
Supplement: Multimedia Appendix 2 [file jmir_v28i1e85973_app2.docx]

| **Item Category** | **Checklist Item** | **Explanation** |
| --- | --- | --- |
| **Design** | Describe survey design | Anonymous survey distributed to all hospitalists at our center, so not considered a convenience sample |
| **IRB (Institutional Review**  **Board) approval and informed**  **consent process** | IRB approval | Considered not to comprise human subjects research by Emory University Institutional Review Board |
|  | Informed consent | First page of survey contained information for consent, if participants consented they would proceed to the survey, else they could decline using the option provided, or simply close the survey. Also had information regarding duration of the survey, that no personal data was collected or stored, how to contact the study investigators, and the reason for the study |
|  | Data protection | Not applicable, no personal information collected or stored |
| **Development and pre-testing** | Development and testing | Developed by discussion and consensus among study authors. Reviewed by division leaders for appropriateness. Functionality was tested prior to dissemination to potential respondents. |
| **Recruitment process and descrip-**  **tion of the sample having access**  **to the questionnaire** | Open survey versus closed sur-  vey | Closed |
|  | Contact mode | Distributed via email to all 70 potential respondents |
|  | Advertising the survey | Reminded potential respondents over a period of 3 weeks via group emails and in-person interactions |
| **Survey administration** | Web/E-mail | Email sent to all potential respondents containing a brief description of the study and a link to the survey itself. The survey was created in Microsoft Forms, that allowed responses to be automatically saved as a Microsoft Excel spreadsheet |
|  | Context | Not applicable |
|  | Mandatory/voluntary | Voluntary |
|  | Incentives | None |
|  | Time/Date | August – September 2025 |
|  | Randomization of items or  questionnaires | No |
|  | Adaptive questioning | Yes |
|  | Number of Items | Maximum number: 17, could be fewer depending on responses |
|  | Number of screens (pages) | Average 2 questions per screen (for readability); maximum 9 screens |
|  | Completeness check | All questions were marked mandatory, so incomplete responses were not allowed |
|  | Review step | Respondents could adjust their responses before submitting, at which time further editing of responses was not allowed |
| **Response rates** | Unique site visitor | Not applicable |
|  | View rate (Ratio of unique sur-  vey visitors/unique site visitors) | Not applicable |
|  | Participation rate (Ratio of  unique visitors who agreed to  participate/unique first survey  page visitors) | Not applicable |
|  | Completion rate (Ratio of users  who finished the survey/users  who agreed to participate) | Not applicable |
| **Preventing multiple entries from**  **the same individual** | Cookies used | No |
|  | IP check | No |
|  | Log file analysis | No |
|  | Registration | Unique responses were ensured by requiring respondents to log in (automatically fulfilled when accessing the survey through clicking the link in their email), survey not accessible after initial completion |
| **Analysis** | Handling of incomplete ques-  tionnaires | Not applicable |
|  | Questionnaires submitted with  an atypical timestamp | No specific analysis |
|  | Statistical correction | No weighting or propensity scores used |
|  |  |  |
