# Supplementary figures and images for: Patterns of AI Use in Clinical Work by Hospitalists: Survey Study
Source: J Med Internet Res. 2026 Mar 3;28:e85973. doi: 10.2196/85973 (PMC12996894; doi:10.2196/85973)

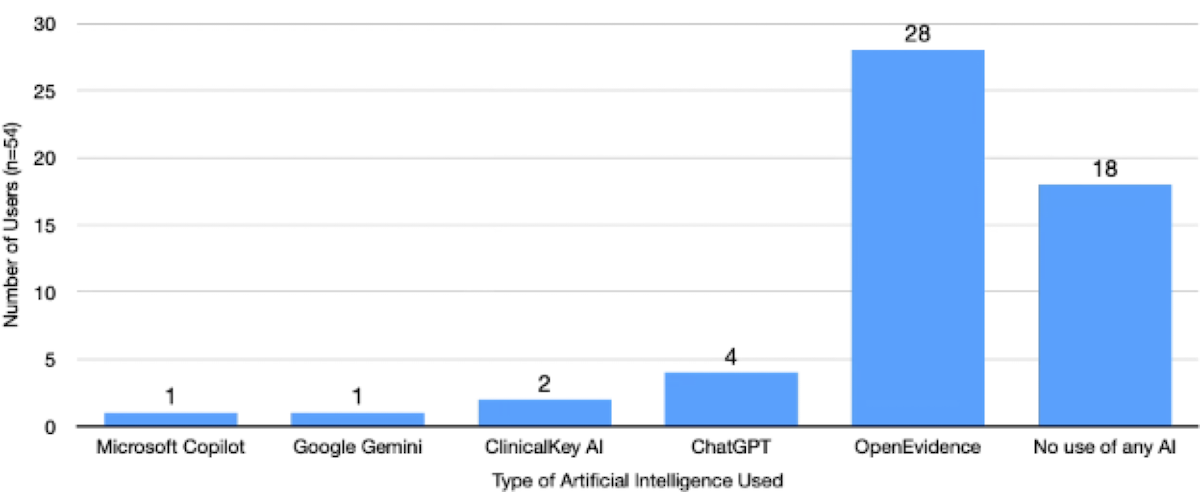

Supplement: Multimedia Appendix 3 [file jmir_v28i1e85973_app3.png]

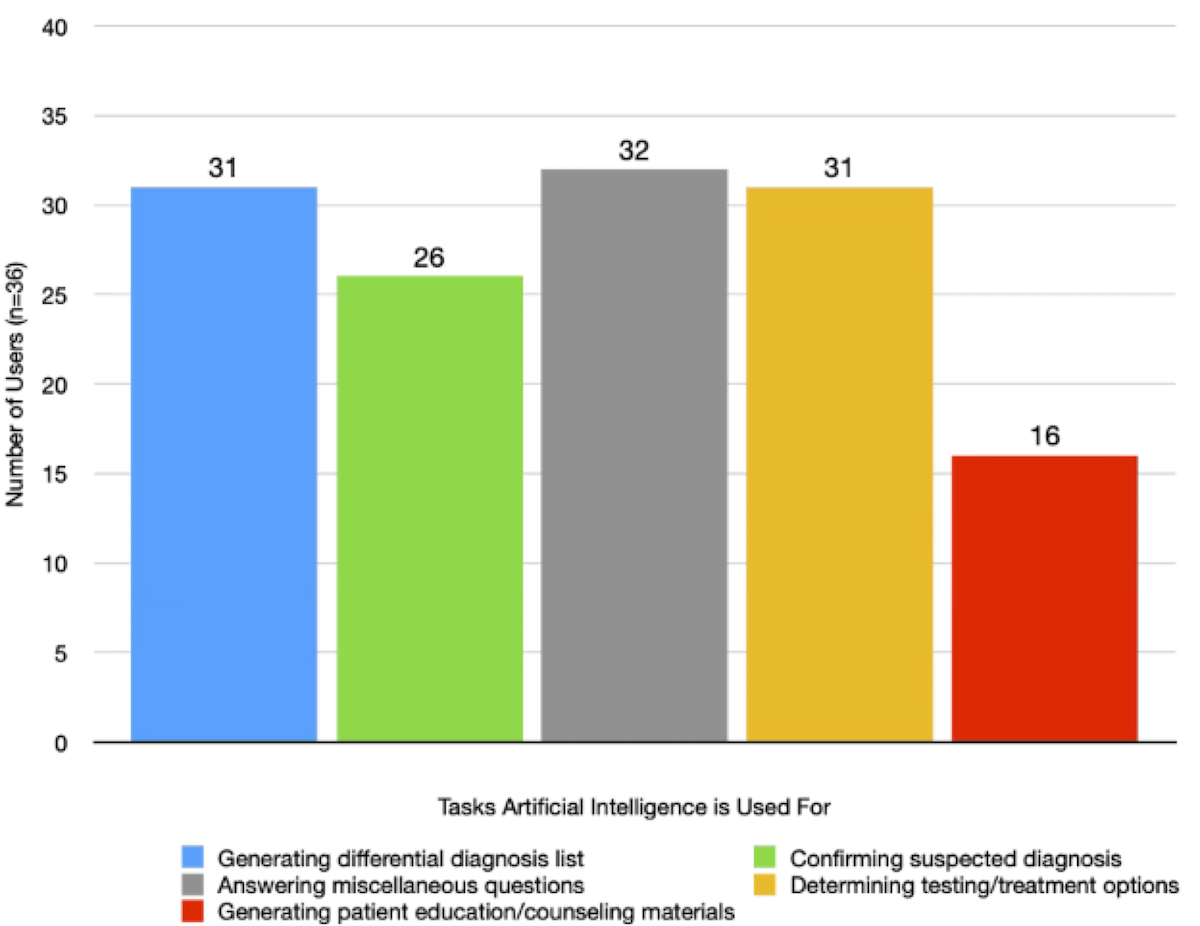

Supplement: Multimedia Appendix 4 [file jmir_v28i1e85973_app4.png]
